# Exceptional Response to Systemic Therapy in Advanced Metastatic Gastric Cancer: A Case Report

**DOI:** 10.7759/cureus.457

**Published:** 2016-01-12

**Authors:** Bradley Colton, Marion Hartley, Maria A Manning, John E Carroll, Joanne Xiu, Brandon G Smaglo, Sameh Mikhail, Mohamed E Salem

**Affiliations:** 1 Oncology, Medstar Georgetown University Hospital; 2 Radiology, Medstar Georgetown University Hospital; 3 Gastroenterology, Medstar Georgetown University Hospital; 4 Oncology, Caris Life Sciences; 5 Oncology, The Ohio State University-James Cancer Hospital and Richard Solove Research Institute

**Keywords:** gastric cancer, molecular profiling, thymidylate synthase, ercc1, topoisomerase 1, cancer biomarkers, esophagogastric adenocarcinoma, cancer treatment, her2

## Abstract

Gastroesophageal adenocarcinomas represent one of the top five most common types of cancer worldwide. Despite significant advancement, it is still not known which first-line chemotherapy option is best matched to an individual patient. The vast advances in molecular biology have led to the discovery of many potential predictive biomarkers, such as HER-2 neu, thymidylate synthase (TS), excision repair cross-complementation group 1 (ERCC1), and topoisomerase-1 (TOPO1). These markers could allow us to select treatment based on an individual’s tumor profile, resulting in an improvement of outcome. Our report highlights two patients with metastatic gastric cancer that achieved an exceptional response with traditional therapy and provides insights into the future perspectives of molecular profile-directed chemotherapy.

## Introduction

Gastroesophageal cancer remains one of the most common forms of cancer worldwide. In 2014, it was estimated that there would be 24,590 and 16,980 new cases of gastric and esophageal cancers, respectively, and 26,310 deaths from gastroesophageal cancer in the United States alone [1]. 

Despite significant advancement in the treatment of metastatic gastric cancer and improvements in patient survival, it is still not known which first-line chemotherapy option is best matched to an individual patient. In other words, the decision to treat any specific patient with first line FOLFOX (folinic acid, fluorouracil, and oxaliplatin), FOLFIRI (folinic acid, fluorouracil, and irinotecan), ECF (epirubicin, cisplatin, and fluorouracil), DCF (docetaxel, cisplatin, and fluorouracil), or EOX (epirubicin, oxaliplatin, and capecitabine) is subjective and remains largely an empirical decision.

Here, we report on two patients with metastatic gastric cancer who achieved an exceptional response to traditional therapy, highlighting the importance of molecular characterization of tumors to identify potential responders. We also discuss future perspectives on molecular profile-directed chemotherapy.

## Case presentation

Informed patient consent was obtained from both patients prior to treatment. No identifying patient information is contained within this paper.

### Case #1

**Figure 1:**
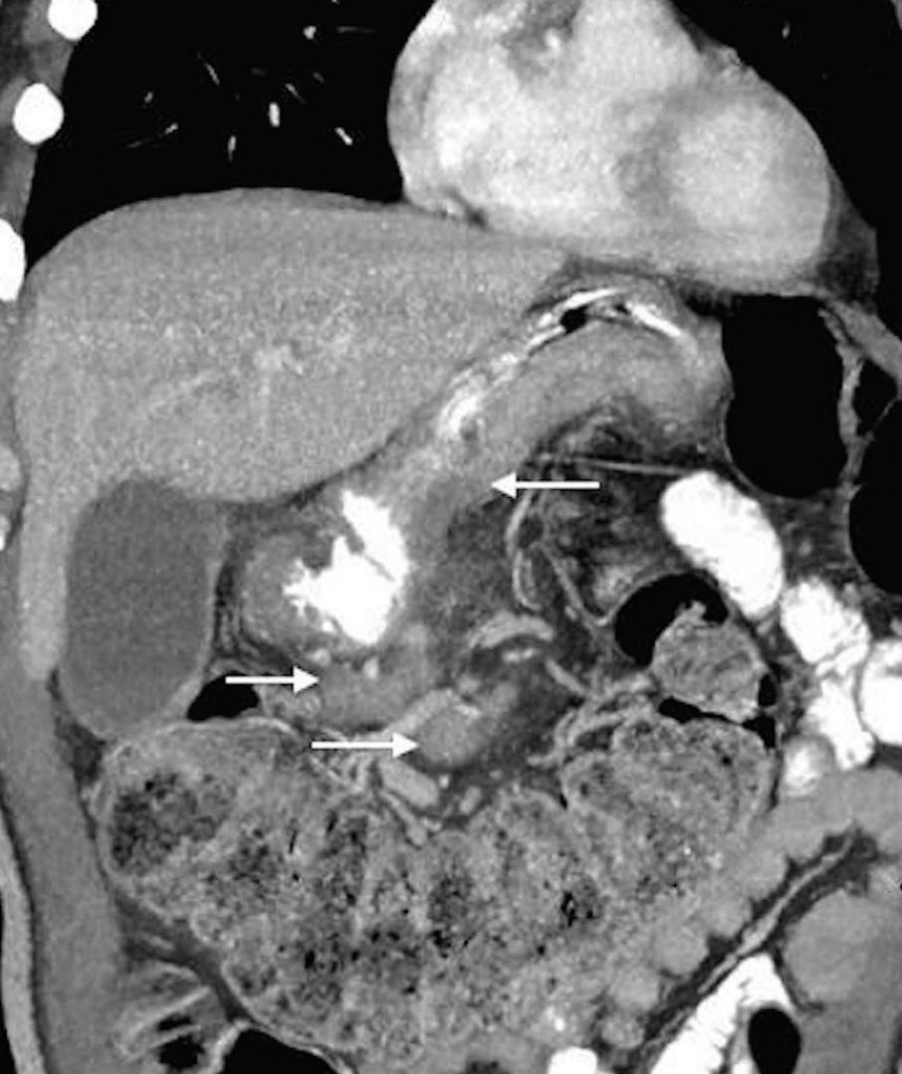
Coronal oblique 5 mm MIP reconstruction CT image from Patient 1 before treatment. This image shows asymmetric, diffuse thickening of the inferior wall of gastric body and antrum with infiltration of adjacent fat as well as extensive mesenteric lymphadenopathy.

**Figure 2:**
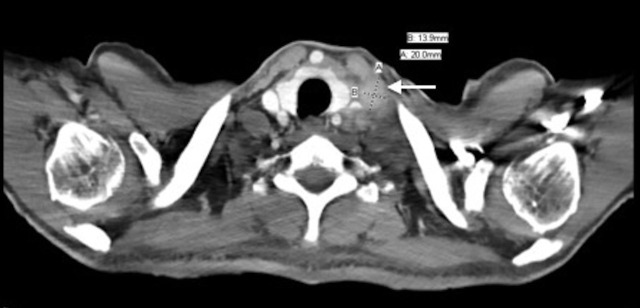
Transverse CT image taken from Patient 1 before treatment. This image shows a left supraclavicular adenopathy measuring 1.4 x 2.0 cm.

A fifty-three-year-old man presented for evaluation regarding his newly diagnosed metastatic adenocarcinoma of the stomach. He reported that he started to experience abdominal and back pain, decreased appetite, weight loss, and fatigue. Upper endoscopy showed a large gastric mass in the body and lesser curvature of the stomach. Biopsy results were consistent with adenocarcinoma. CT scan on July 1, 2015 (Figures 1-2) showed asymmetric, diffuse thickening of the inferior wall of the gastric body and antrum with infiltration of adjacent fat, a peritoneal nodule, and extensive mesenteric lymphadenopathy. The scan also revealed left periaortic adenopathy, measuring 1.4 x 2.0 cm, and a retrocaval lymph node, measuring 3.0 x 1.6 cm. The patient was started on systemic chemotherapy with FOLFOX.

**Figure 3:**
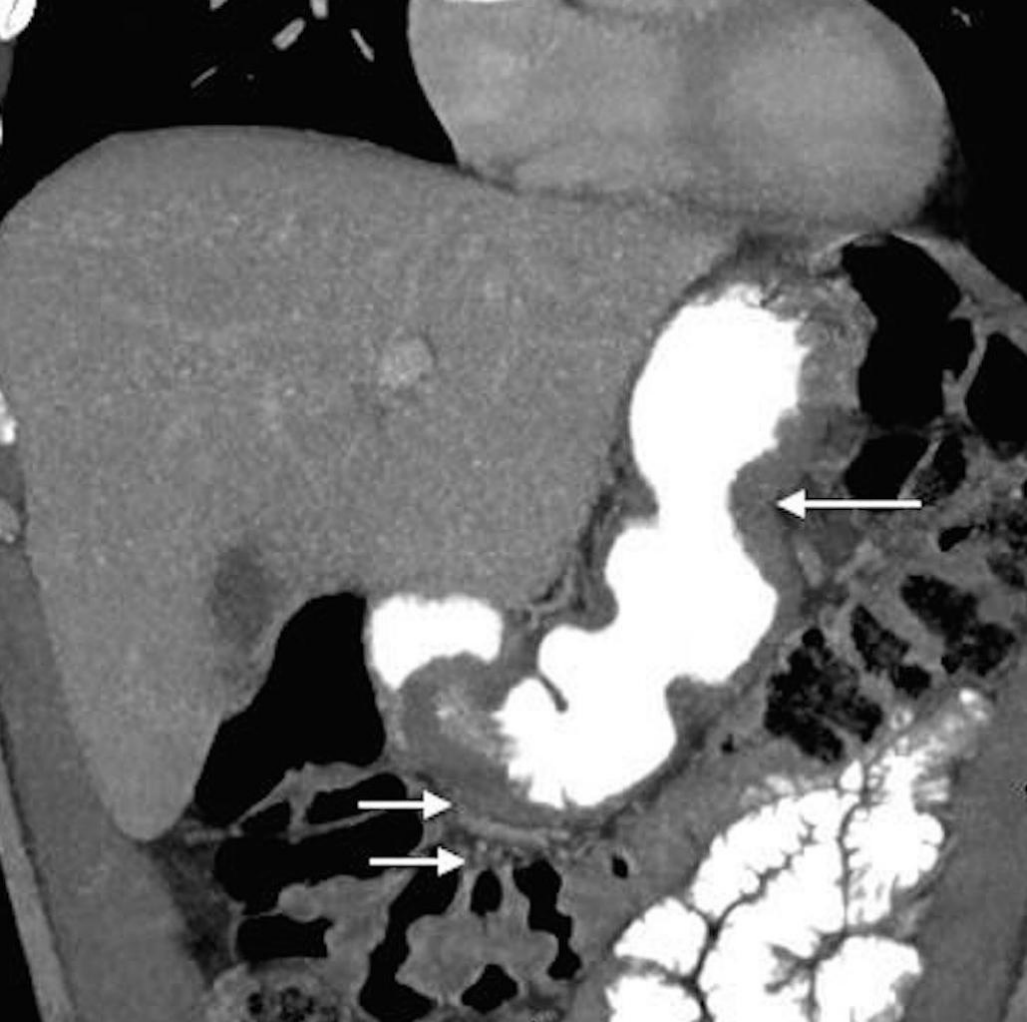
Coronal oblique 5 mm MIP reconstruction CT image taken from Patient 1 after four cycles of FOLFOX. This image shows significant interval decrease in disease burden with a marked decrease in the size of the primary gastric mass in addition to a complete resolution of the previously documented infiltration of adjacent fat and extensive mesenteric lymphadenopathy.

**Figure 4:**
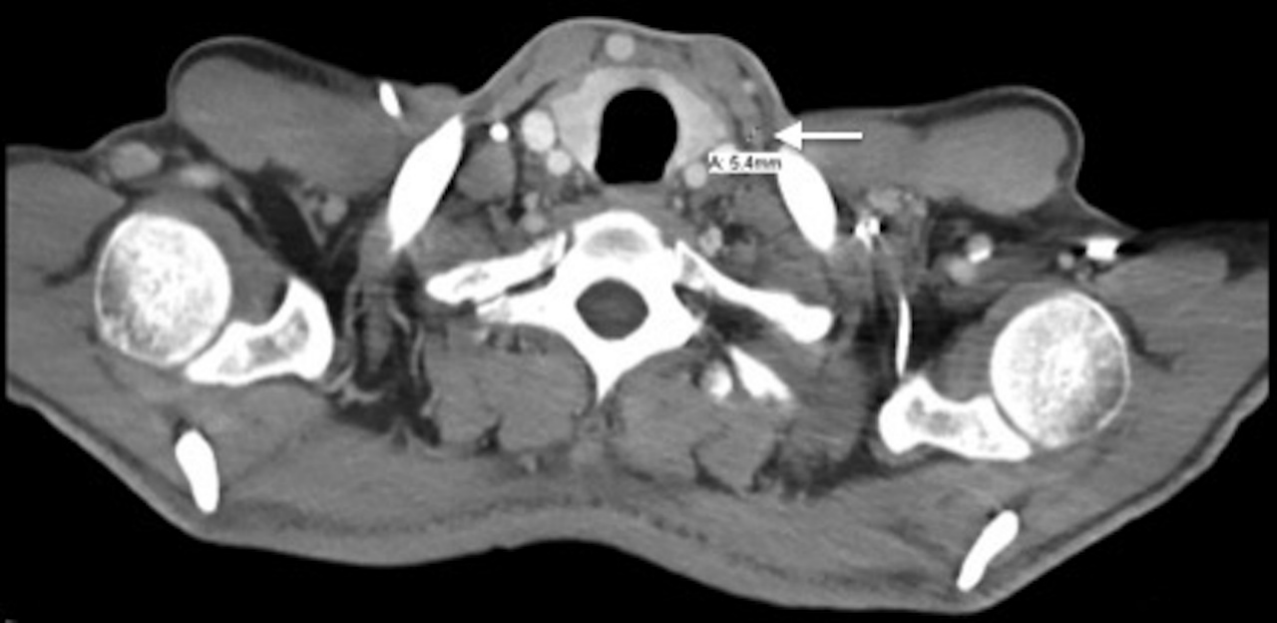
Transverse CT image taken from Patient 1 after four cycles of FOLFOX. This image shows that the left supraclavicular node decreased in size to 0.8 x 0.6 cm from 1.4 x 2.0 cm.

Following four cycles of FOLFOX, the patient underwent a restaging CT (Figures 3-4). The results showed a significant interval decrease in disease burden, with a marked decrease in the size of the primary gastric mass and complete resolution of previously documented infiltration of adjacent fat, peritoneal nodule, and extensive mesenteric lymphadenopathy. It also showed that the left periaortic node had decreased to 0.8 x 0.6 cm and the retrocaval lymph node to 1.2 x 0.7 cm. Clinically, the patient was feeling dramatically better with the resolution of his initial presenting symptoms: he had no complaints and reported that his abdominal and back pain had completely resolved, he had a good appetite, he was gaining weight, and his energy levels were improving.

**Figure 5:**
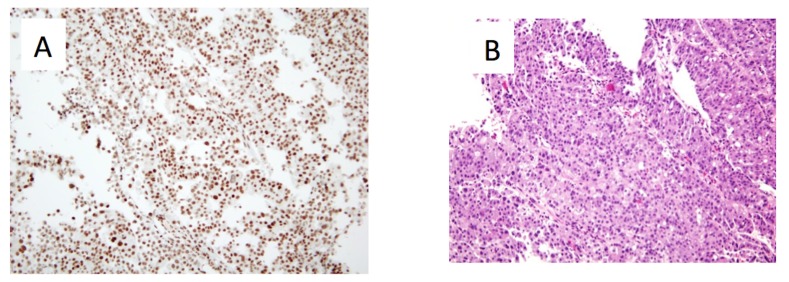
Representative microscopic images of the gastric tumor taken from Patient 1 at a magnification of 20x. A) ERCC1 IHC staining read as intensity 2+ (on a scale of 0-3. 0: no staining; 1+: faint staining; 2+: weak to moderate staining; 3+: strong staining) on 65% of tumor cells. B) Haematoxylin and eosin staining showing poorly differentiated adenocarcinoma.

The patient’s outstanding response prompted us to perform a posthoc tumor profiling analysis to look for underlying molecular aberrations, the knowledge of which may provide insight into the identification of potential responders in the future (Figure 5). Expression of DNA repair pathway genes, ERCC1 and PTEN, were intact (2+, 65% and 1+, 100%, respectively) as shown by immunohistochemistry. NextGen sequencing (Illumina NextSeq platform using an Agilent custom-designed SureSelect XT assay on 591 whole-gene targets) revealed no mutation in the various markers within the homologous recombination (HR) pathway, including BRCA1/2, ATM, CHEK1/2, and PTEN. Interestingly, a truncating mutation on PALB2 (S326*), in addition to a mutation c.3201+2_3201+3insT, was found. PALB2 (Partner and localizer of BRCA2) plays an important role in homologous recombination of DNA (double-strand break) repair. Mutations in PALB2 have been previously reported in gastric cancer [2], but their therapeutic implications have not yet been explored. In various other cancer types, homologous recombination deficiencies, indicated by aberrations on the HR pathway, including PALB2 mutations, have been associated with increased sensitivity to platinum treatments and, therefore, prolonged survival [3]. Thus, it is likely that the PALB2 mutation in the tumor of the patient in the current report led to the loss of function of PALB2 and the observed outstanding response to oxaliplatin-based chemotherapy.

### Case #2

**Figure 6:**
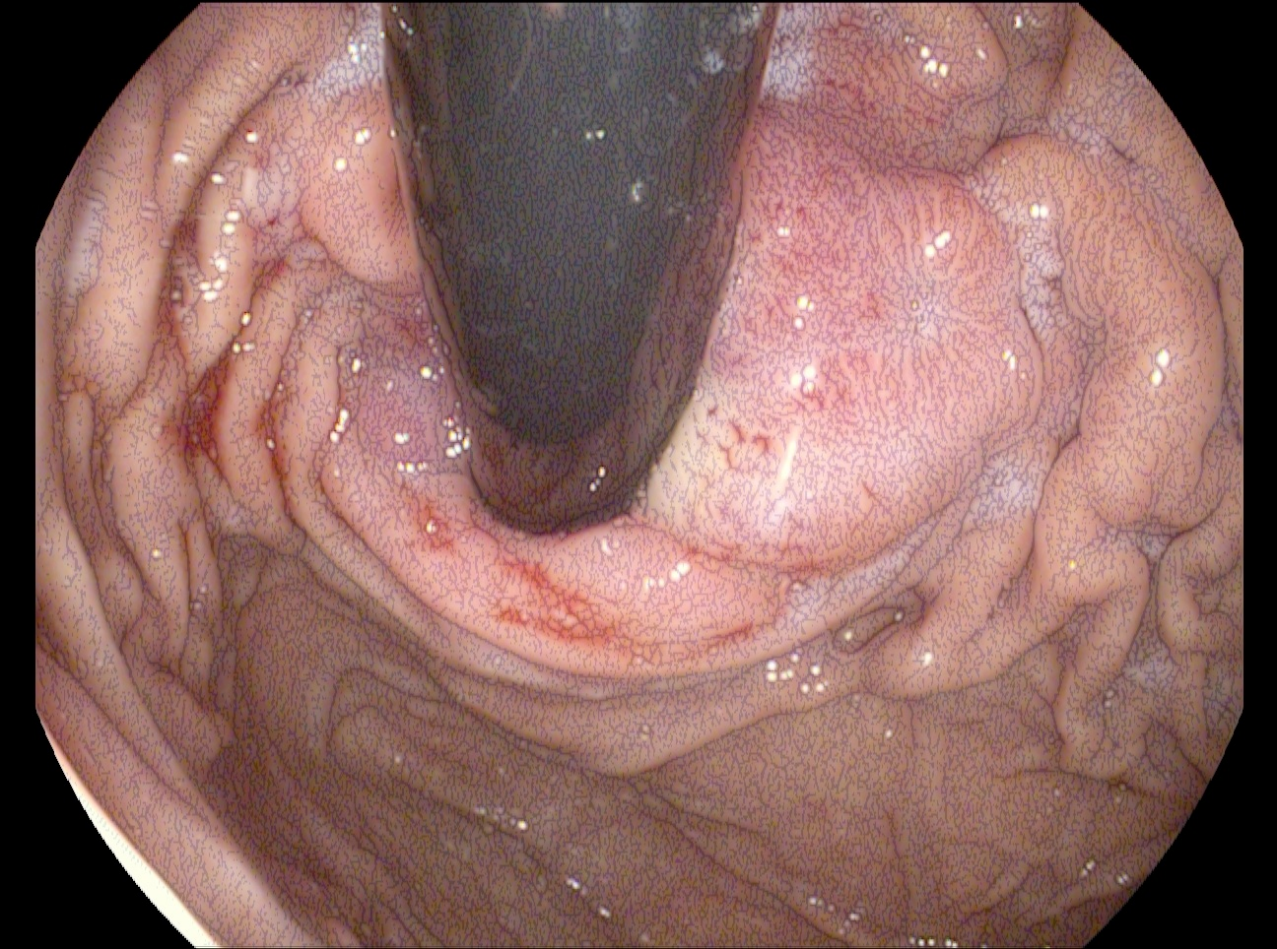
Image from esophagogastroduodenoscopy (EDG) performed on Patient 2 before she received therapy. This image shows a tight, friable, fungating, malignant-appearing mass circumferentially involving the gastric cardia and gastroesophageal valve.

**Figure 7:**
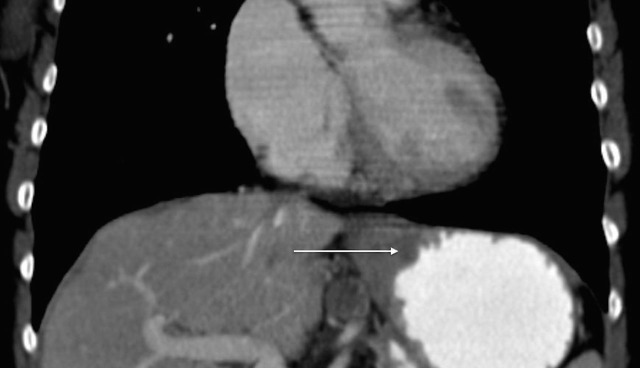
Contrast-enhanced CT image of the abdomen from Patient 2 prior to treatment. This image reveals asymmetric wall thickening of gastric cardia, measuring approximately 2.7 x 4 cm, with stranding into adjacent fat.

A 68-year-old Caucasian woman was referred to Georgetown University Hospital for evaluation of her newly diagnosed adenocarcinoma of the gastric cardia. She reported that she first noticed increased belching in the spring of 2014. This symptom progressed and her appetite worsened; she had to eat smaller, more frequent meals and had one episode of emesis after a larger meal. Subsequently, an endoscopy was performed revealing a large mass at the gastric cardia (Figure 6). Biopsy revealed moderately differentiated adenocarcinoma. Further assessment by CT showed asymmetric wall thickening of the gastric cardia measuring approximately 2.7 x 4 cm, with stranding into adjacent fat. Gastrohepatic ligament lymphadenopathy, measuring 1.8 x 1.3 cm, was also observed, along with about five subcentimeter indeterminate liver lesions (Figure 7) .

**Figure 8:**
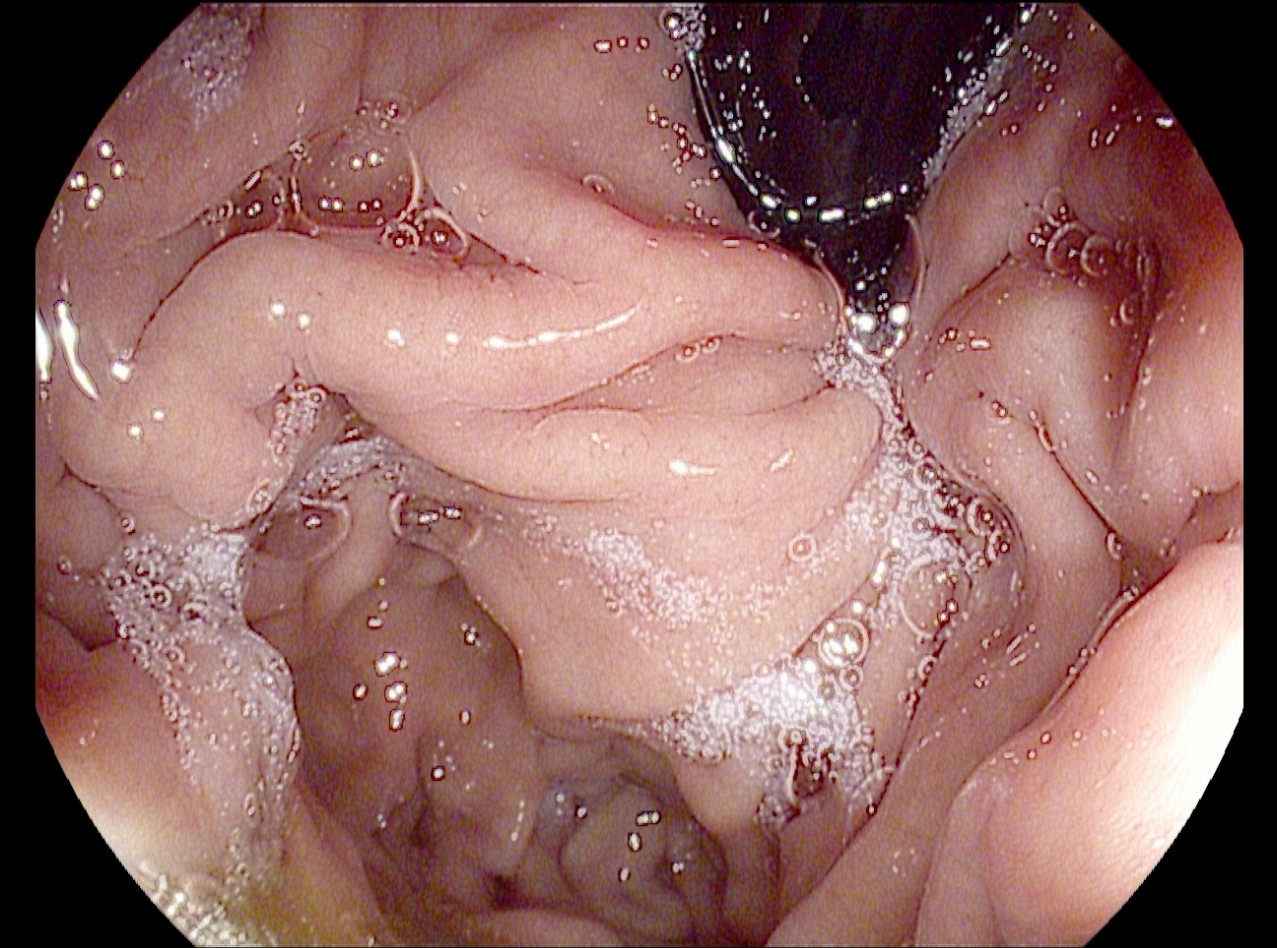
Image from EDG performed on Patient 2 after six cycles of EOX and four months of capecitabine monotherapy. This image shows normal-appearing gastric cardia without endoscopic evidence of disease.

**Figure 9:**
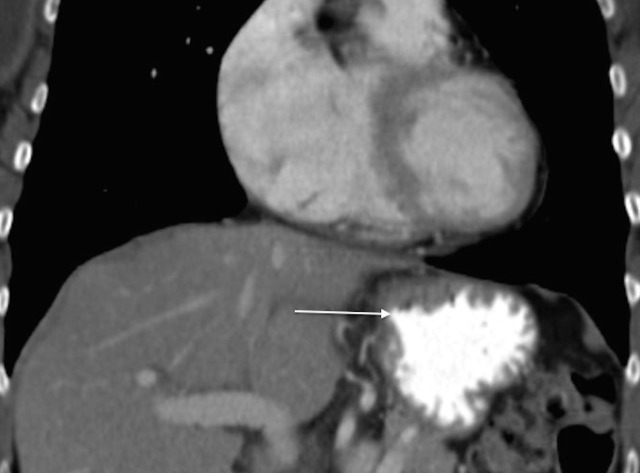
Contrast-enhanced CT image of the abdomen from Patient 2 after continued treatment with capecitabine monotherapy. This image shows continued absence of disease, with no appreciable mass in the gastric cardia.

The patient proceeded with systemic chemotherapy, starting with EOX. After just two cycles of therapy, there was significant tumor response with no discrete mass seen in the gastric cardia, the disappearance of the liver lesions, and no change is the size of the gastrohepatic lymph node. After six cycles of EOX, CT imaging revealed a continued response to therapy; there was no mass appreciated in the gastric cardia, a reduction in the size of the gastrohepatic ligament lymphadenopathy (down to 1.3 x 1.3 cm), and no evidence of hepatic lesions. Treatment continued with capecitabine monotherapy. Follow-up esophagogastroduodenoscopy (EGD) showed normal gastric cardia with no endoscopic evidence of disease (Figure 8). At the patient’s last visit in November 2015, she showed no evidence of disease by CT scan, with the exception of a stable, subcentimeter, gastrohepatic ligament lymph node (Figure 9). Unfortunately, molecular profile results of this tumor could not be obtained.

## Discussion

### Treatment of gastroesophageal adenocarcinoma

Chemotherapy is the standard therapy for metastatic esophagogastric adenocarcinoma. The REAL-2 trial showed that the toxicity profile was better in patients who received capecitabine-based regimens, such as EOX (epirubicin and oxaliplatin, combined with capecitabine) [4]. Thus, oxaliplatin and capecitabine have often been substituted for cisplatin and 5-FU.

The REAL-2 trial showed that the toxicity profile in patients who received capecitabine-based regimens, such as EOX, was the best of all viable chemotherapeutic options [5].

There is now a range of anticancer agents available for gastroesophageal adenocarcinomas. However, the choice of treatment has always been rather empiric, and it is unknown whether a more effective targeted treatment exists for this cancer type. Hence, remarkable efforts are currently ongoing to establish a new treatment strategy that will enable us to better select patients and deliver a precise therapy for any particular individual based on their specific tumor profile.

### Molecular profiling and potential predictive biomarkers

Molecular profiling of tumors from patients with advanced gastric cancer has recently been an area of intense investigation; whether molecular profile-guided therapy can result in an improvement of outcome remains an unanswered question and is currently being addressed in several trials.

### HER2

One successful example of molecular targeted therapy in the management of gastric cancer is the use of the anti-HER2 monoclonal antibody (mAb), trastuzumab (Herceptin, Genentech). Using FISH and IHC techniques, HER2 overexpression has been discovered in adenocarcinomas of the gastroesophageal (GE) junction. The ToGA trial validated the role of a trastuzumab chemotherapy combination as a new standard treatment for patients with HER2(+) gastroesophageal cancers. This study showed a significantly higher objective response rate in HER2+ patients when adding trastuzumab to standard chemotherapy (47%) compared with standard chemotherapy alone (35%). The median overall survival was also shown to be significantly better with the addition of trastuzumab (13.8 vs. 11.1 months) [6].

### Thymidylate synthase (TS)

Thymidylate synthase (TS) is the target enzyme for 5-fluorouracil (5-FU). Johnston, et al. illustrated how patients with disease responsive to 5-FU had TS protein levels that were significantly lower than those in patients with unresponsive disease (mean ± SD = 0.17 ± 0.03 vs. 0.60 ± 0.09; P < 0.01). A similar pattern was noted with TS gene expression. TS protein and TS mRNA expression were highly correlated, and both predicted for response to 5-FU + LV-based chemotherapy in patients with gastric and colorectal cancers: low TS protein and mRNA levels predicted a positive treatment response, whereas high levels predicted treatment failure [7]. Another study by Alexander, et al. evaluated TS tumor protein expression and its association with response to neoadjuvant chemotherapy and patient survival. Twenty patients with gastric or gastroesophageal adenocarcinoma were treated with neoadjuvant fluorouracil, leucovorin, and interferon alpha, followed by resection and three cycles of consolidation therapy. Subsequent analyses showed that pretreatment levels of total thymidylate synthase were significantly higher in non-responders (n = 5) than in responders (n = 8). This study indicated that a response to neoadjuvant fluorouracil-based therapy might be associated with lower levels of total thymidylate synthase before therapy [8].

### DNA repair defects

DNA interstrand crosslinks caused by platinum agents are repaired by a sequential combination of DNA repair mechanisms, including nucleotide excision repair (NER) and homologous recombination (HR); single-strand DNA breaks that fail to be repaired by base-pair repair (BPR) mechanisms can eventually become double-strand breaks, which are then repaired by HR. Low expression levels of ERCC1, a structure-specific endonuclease that plays an essential role in NER-mediated DNA repair, have been associated with platinum drug sensitivity. Fareed, et al. performed a study where formalin-fixed human gastroesophageal cancers were constructed into tissue microarrays. One set consisted of 142 cases that were not exposed and the second set consisted of 103 cases that were exposed to preoperative platinum-based chemotherapy. Expression of ERCC1, XPF, FANCD2, APE1, and p53 were subsequently measured using immunohistochemistry. ERCC1 nuclear expression correlated with a lack of histopathological response to neoadjuvant platinum-based chemotherapy (P = 0.006) and was associated with poor disease-specific (P = 0.020) and overall (P = 0.040) survival [9]. This study provides evidence that ERCC1 nuclear protein expression may be a promising predictive marker for response of gastroesophageal cancers to platinum-based chemotherapy. While it has been shown that HR deficiency plays an important role in the progression of gastric carcinoma, this privation also renders the cancer cells unable to repair damages caused by platinum agents and confer drug sensitivity. Various studies involving patients with NSCLC, bladder cancer, ovarian cancer, and triple negative breast cancer have shown that molecular aberrations on the HR pathway, including BRCA1, BRCA2, ATM, BARD1, BRIP1, CHEK1/2, PALB2, and RAD51C/D, are associated with a significant prolongation of overall survival in individuals treated with platinum agents [3]. Overall, a comprehensive survey of DNA repair pathways may help to identify patients who can potentially benefit from DNA damaging agents, including platinums.

### Topoisomerase-1 (TOPO1)

Topoisomerase-1 (TOPO1) expression in tumors has been shown to correlate with irinotecan response in several large studies. Depending on “high” or “low” expression, TOPO1 was shown to be a predictive marker associated with the benefit of irinotecan and oxaliplatin, as well as a prognostic marker associated with outcomes following 5-FU therapy alone. It was found that patients with low TOPO1 levels responded relatively well to first-line 5-FU treatment, but did not benefit in terms of PFS and overall survival benefit from the addition of either irinotecan or oxaliplatin to their regimen. With increasing TOPO1 expression, the outcome following 5-FU treatment alone was worse, but the addition of a second drug became worthwhile, with a major improvement in survival for the highest TOPO1-expressing patients on combination therapy [10].

## Conclusions

Vast advances in molecular biology have led to the discovery of many potential predictive biomarkers of carcinogenesis in organ systems throughout the human body. Patients with a similar diagnosis can have dramatically different responses to the same treatment. Whereas one patient may have no benefit at all, others, such as seen in the two patients reported in this paper, can have impressive and unexpected remissions. For the patient case 1, the posthoc analysis revealed the presence of a PALB2 mutation that likely led to the outstanding response to oxaliplatin-based therapy. It would have been interesting to obtain molecular profiling results for patient case 2 in order to investigate a similarly impactful molecular aberration that could be responsible for the dramatic response of this patient’s tumor to chemotherapy. However, this was not possible.

Theoretically, the collection of pre-treatment tumor biopsies and subsequent molecular profiling could define biomarkers that may then allow us to select treatments for patients on an individual basis and improve disease outcomes. This approach is being investigated right now in a number of different cancers and, if proven effective, will result in a paradigm shift to a “tailor-made” therapy that can offer an unprecedented opportunity to improve the current paradigm. Extended biomarker validation will enable further tailoring of cancer therapy to individual patients and their tumors moving forward.
